# Very-low-calorie-diet, tirzepatide, and bariatric surgery: a multidisciplinary success in super-super obesity

**DOI:** 10.1093/jscr/rjae816

**Published:** 2025-03-28

**Authors:** Carla Daou, Juan S Barajas-Gamboa, Cynthia Salloum, Alfredo Daniel Guerron

**Affiliations:** Digestive Disease Institute, Department of General Surgery and Center for Medical Nutrition, Al Falah St, Al Maryah Island, Abu Dhabi 112412, United Arab Emirates; Digestive Disease Institute, Department of General Surgery and Center for Medical Nutrition, Al Falah St, Al Maryah Island, Abu Dhabi 112412, United Arab Emirates; Medical Subspecialties Institute, Department of Endocrinology, Al Falah St, Al Maryah Island, Abu Dhabi 112412, United Arab Emirates; Digestive Disease Institute, Department of General Surgery and Center for Medical Nutrition, Al Falah St, Al Maryah Island, Abu Dhabi 112412, United Arab Emirates

**Keywords:** super-super obesity, multidisciplinary team, bariatric surgery, nutrition, medications

## Abstract

This case report highlights the multidisciplinary team (MDT) approach in managing a 49-year-old male with super-super obesity (initial Body Mass Index (BMI): 106.25 kg/m^2^). Interventions included very-low-calorie-diet, tirzepatide treatment, and eventual sleeve gastrectomy. Pre-operatively, the MDT focused on optimizing the patient’s metabolic status and surgical readiness. The patient, initially bedbound with comorbidities including type 2 diabetes and obstructive sleep apnea, achieved significant weight loss pre-surgery. Post-operatively, the patient followed bariatric diet phases and engaged in physical therapy, improving mobility from bedbound to walking 25 minutes with a walker. Over 1 year, the patient achieved 37% excess weight loss, reducing BMI to 66.79 kg/m^2^. This case demonstrates the effectiveness of an MDT approach in treating super-super obesity, leading to substantial weight loss and improved health outcomes.

## Introduction

Obesity severity is often categorized using Body Mass Index (BMI), with super-obesity defined as BMI >50 kg/m^2^ and super-super obesity as BMI >60 kg/m^2^ [1]. These extreme forms of obesity, while rare, present significant clinical challenges due to associated complex comorbidities.

Obesity treatment includes bariatric surgery procedures [2] and, more recently, glucagon-like peptide-1 receptor agonists (GLP-1 RAs) for pharmacological management [3]. However, managing super-super obesity remains exceptionally challenging due to increased surgical risks, limited effectiveness of standard pharmacotherapy, and complex psychosocial factors. These challenges necessitate a comprehensive, multidisciplinary team (MDT) approach [4].

While MDT approaches have shown efficacy in obesity management, detailed guidance and well-documented long-term outcomes for super-super obesity cases (BMI >60 kg/m^2^) are lacking, partly due to the infrequency of such cases [4]. This knowledge gap underscores the importance of reporting successful management strategies for these complex patients.

In this case report, we present the successful management of a patient with extreme super-super obesity (initial BMI of 106.25 kg/m^2^) using a comprehensive MDT approach. Our strategy combined nutritional intervention through a very-low-calorie diet, pharmacological treatment with tirzepatide, and surgical management via sleeve gastrectomy, demonstrating the potential of an integrated approach in managing such challenging cases.

## Case Report

### Admission and in-patient management

On 25 October 2023, a 49-year-old male presented to the bariatric clinic seeking bariatric surgery. The patient had an initial weight of 272 kg (BMI 106.25 kg/m^2^) and a history of type 2 diabetes mellitus (T2DM), obstructive sleep apnea (OSA) requiring nocturnal BiPAP, bilateral lipedema, and had been bedbound for 2 years. Given the patient’s poor overall health condition and lack of social support, a decision was made to admit him for comprehensive evaluation and optimization. MDT assessment included pulmonology, cardiology, nutrition, endocrinology, psychiatry, physical therapy (PT), and wound care.

The MDT collectively determined that the patient required preoperative optimization before considering laparoscopic sleeve gastrectomy (LSG). The primary goals were to achieve ~10% initial weight loss through nutritional management and to improve mobility and lean mass through physical therapy. The patient’s nutritional management began with a liver reducing diet (LRD) of 800 calories daily for the first 3 weeks of admission, supplemented with multivitamins and iron. On 10 November 2023, the diet was adjusted to a very low-calorie diet (VLCD) of 800–1100 calories per day, tailored daily by a registered dietitian (RD).

Concurrently, the endocrinology team initiated tirzepatide at 2.5 mg, with dose escalation to 5 mg after 1 month. The dosage was then increased by 2.5 mg every 2 weeks, deviating from standard recommendations of 4-week intervals, due to concerns about absorption at lower doses. This regimen was well-tolerated without adverse effects. Due to medication shortages, tirzepatide was temporarily discontinued in January 2024 for one month. It was restarted in February 2024 at 5 mg, gradually increased to 10 mg by 25 March 2024, and then discontinued 2 weeks prior to the scheduled LSG on 8 April 2024.

As for the diet, patient remained on VLCD throughout admission until 2 weeks pre-LSG when he was placed again on LRD, taking three meal replacements daily. By 25 April, the patient had lost 70 kg to reach 202 kg (78.9 Kg/m^2^) pre-LSG and this is when he underwent LSG. Table 1 shows the trend of nutrition related laboratory values that were checked throughout the patient’s stay.

**Table 1:** Laboratory parameters trend

Date	Laboratory Parameter	Vitamin D (nmoL/L)	Vitamin A (μmoL/L)	Vitamin E (μmoL/L)	Iron (micromol/L)	Folate (nmoL/L)	HbA1C (%)	Pre-albumin (g/L)
25/10/23		24	0.37	12	4.2	27.1	8.80	0.05
3/1/2024		59	0.82	38	5.1	Not checked	Not checked	0.11
24/2/2024		Not checked	0.72	32	4.7	31.5	5.60	0.10
Post-op–5/8/2024		Not checked	Not checked	29	6.8	Not checked	5.30	0.12

### Post-operative management

**Figure 1:**
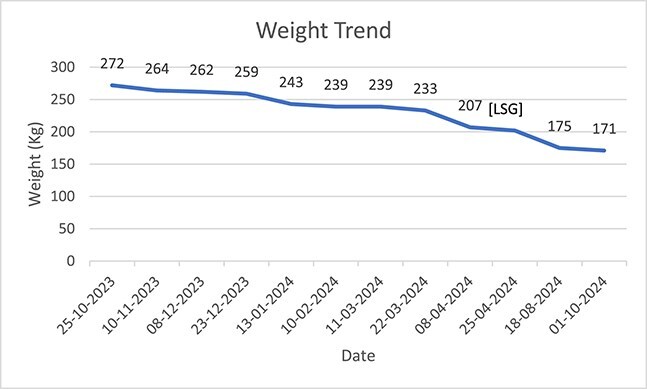
Weight trend since admission until October 2024, pre and post-op.

**Figure 2:**
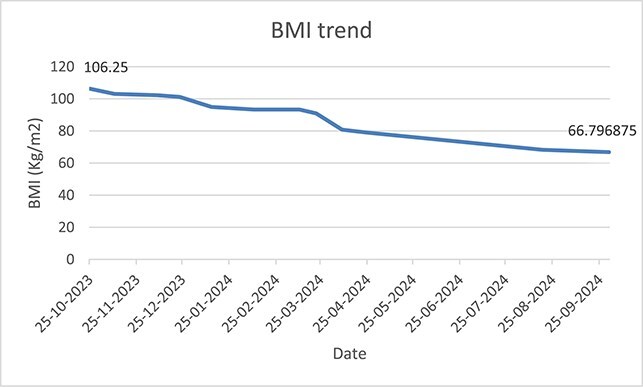
BMI trend since admission until October 2024, pre and post-op.

**Figure 3:**
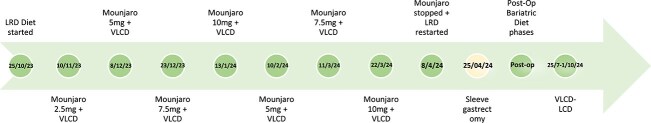
Time line reflecting treatment care for throughout admission.

Patient continued to receive comprehensive care via MDT approach. Patient was initiated on the standard bariatric diet phases post-op in which he followed bariatric diet phases 1 to 5 for 1.5 months post-op and then was kept on VLCD afterwards until October 2024. RD was ordering patient’s meals daily based on a calorie count diet. Additionally, PT helped patient ambulate as he managed to stand up for the first time 1-month post-op and progressed his ambulation distance by walking 25 m using a walker. Psychology and social worker teams also supported patient by tackling behavioral challenges, mental health and access to necessary resources. By October 2024, patient weighed 171 kg (BMI 66.79 Kg/m^2^), and a total percentage excess weight loss (%EWL) of 37%. Figures 1 and 2 show the weight and BMI trend throughout admission. Figure 3 shows the timeline of the treatment care. As of that date, patient was transferred to long term rehabilitation unit and was moved under rehabilitation team.

## Discussion

This case report demonstrates the successful management of a super-super obese patient through a MDT approach at our institution, which was notably the only facility in the UAE and Middle East and North Africa (MENA) region with the capacity and resources to treat this complex case, after multiple rejections from other healthcare institutions. The patient’s weight decreased significantly from 272 kg (BMI 106.25 kg/m^2^) to 175 kg (BMI 68.36 kg/m^2^) in 12 months, achieving a total %EWL of 37%. This substantial weight reduction highlights the potential effectiveness of combining dietary, pharmacological, and surgical interventions in managing extreme obesity cases.

Our findings align with previous studies on obesity management strategies. Nauck *et al.* (2021) [5] reported the effectiveness of GLP-1 receptor agonists in managing diabetes in obese patients, while Jensen *et al.* (2014) [6] highlighted the role of VLCD in reducing HbA1c levels pre-bariatric surgery. In our case, we observed a significant HbA1c reduction from 8.8% to 5.6% through the combined use of VLCD and tirzepatide before the LSG. This outcome supports the recommendations of Puzziferri *et al.* [7], who emphasized that combining surgical interventions with intensive lifestyle modifications and pharmacological treatments leads to more substantial weight loss and improved long-term health outcomes compared to individualized treatments. Our case uniquely demonstrates the successful application of this integrated approach in a patient with extreme super-super obesity (BMI >100 kg/m^2^), a population often excluded from clinical trials.

The patient’s progress from being bedbound to walking 25 m with a walker underscores the critical role of physical therapy in managing super-super obese patients. This improvement in mobility not only enhances quality of life but also potentially reduces the risk of complications associated with immobility, such as deep vein thrombosis and pressure ulcers. Furthermore, the normalization of several nutritional parameters, including Vitamins D, A, and E levels, demonstrates the efficacy of our comprehensive nutritional intervention [8]. These improvements in nutritional status are particularly significant given the high prevalence of micronutrient deficiencies in severely obese patients and their potential impact on post-surgical outcomes.

While our approach showed positive outcomes, it had certain limitations. This report covers only 12 months of the patient’s journey, and longer-term outcomes are yet to be determined. Additionally, intermittent unavailability of higher doses of tirzepatide may have affected the consistency of treatment. More frequent monitoring of nutrition-related laboratory values could have provided a more comprehensive picture of the patient’s metabolic changes.

Despite these limitations, our MDT approach demonstrated several strengths. The integration of various specialties allowed for holistic patient care, while the ability to adjust treatment plans based on the patient’s progress and medication availability showcased flexibility and personalized care. The significant pre-surgical weight loss and diabetes management likely contributed to improved surgical outcomes, and intensive monitoring through regular follow-ups ensured patient safety and optimized outcomes [9].

In conclusion, this case report demonstrates the potential of integrated care in managing super-super obesity. The significant improvements in weight, glycemic control, and mobility underscore the importance of personalized, multidisciplinary care in achieving meaningful results for these complex patients. Our findings contribute to the limited body of evidence on successful management strategies for patients with BMI >100 kg/m^2^ and may inform future approaches to treating extreme obesity cases.
